# Surgical timing and clinical factor predicting in-hospital mortality in older adults with hip fractures: a neuronal network analysis

**DOI:** 10.1186/s10195-025-00846-x

**Published:** 2025-05-14

**Authors:** Raffaele Vitiello, Elisa Pesare, Giacomo Capece, Emidio Di Gialleonardo, Andrea De Matthaeis, Francesco Franceschi, Giulio Maccauro, Marcello Covino

**Affiliations:** 1 Agostino Gemelli University Policlinic IRCCS, Rome, Italy; 2 School of Medicine, Department of Basic Medical Sciences, Neuroscience and Sense Organs, Orthopedic and Trauma Unit, University of Bari Aldo Moro, Bari, Italy; 3 Catholic University of The Sacred Heart, Agostino Gemelli University Policlinic IRCCS, Rome, Italy

**Keywords:** Hip fractures, Neuronal network analysis, Sepsis, Older adults

## Abstract

**Introduction:**

Hip fractures in older adults are associated with a significant mortality rate, which has been reported to be around 35% within a year. Today, the incidence of these fractures is on the rise, and this trend is expected to increase even more owing to the aging of the population. Treatment timing and perioperative management of these patients are typically challenging owing to the presence of multiple comorbidities that are important risk factors for mortality after surgery.

This study aims to evaluate the relationship between surgical timing and in-hospital mortality, analyzing the role of both acute events and chronic preexisting comorbidities in patient outcomes.

**Materials and methods:**

This is a single-center, retrospective observational study (from January 2018 until June 2023). All consecutive patients ≥ 65 years with a diagnosis of proximal femur fracture were enrolled. The primary study endpoint was to evaluate risk factors associated with in-hospital mortality. The secondary endpoint was the assessment of the relationship between surgical timing and in-hospital mortality, including factors such as preexisting comorbidities, the Charlson Comorbidity Index, and the Nottingham Hip Fracture Score. The relative weight of each factor for predicting the mortality rate was also evaluated using neural network analysis, comparing patients treated within 24 h to those treated after a longer surgical delay.

**Results:**

Among the 2320 patients enrolled, 1391 (60%) underwent surgery within 24 h, while 929 patients (40%) were treated after 24 h. For patients who underwent surgery within 24 h, the in-hospital mortality was 2.8%, and for those who underwent surgery after 24 h, it was 5.2% (*p* = 0.046; odds ratio (OR) 1.58). Age (*p* = 0.001; OR 1.06) and Nottingham score (*p* = 0.04; OR 1.32) are factors predicting mortality. Acute infections were related to a high risk of mortality (*p* = 0.001; OR 5.99), both in patients treated within and after 24 h. Acute events, such as atrial fibrillation and electrolyte imbalance, were related to mortality risk only in patients treated within 24 h (*p* = 0.001 versus *p* = 0.51). Neural network analysis revealed that atrial fibrillation (AF), flutter, and electrolyte imbalance had the highest relative weight for mortality in patients treated in the first 24 h; by contrast, renal failure and pneumonia were most present in patients who died that were treated after 24 h.

**Conclusions:**

Hip fracture is known to be a significant cause of morbidity and mortality in older adults. The impact of the timing of surgical treatment in those patients is crucial for postoperative outcomes. Early surgery is essential to reduce the risk of mortality. Our study has shown that, while in the case of acute and reversible conditions, waiting about 24 h to stabilize the patient with preoperative stabilization protocols, such as managing anticoagulation, optimizing hemodynamics, or addressing acute medical conditions including infection prevention, guarantees better results, in the case of sepsis or acute infection presence, the prolonged waiting to optimize patients before and after surgery does not help improve outcomes.

## Introduction

Hip fractures are the most common orthopedic injury in older adults and osteoporotic patients [1]. This condition is well-known to be related to a significant mortality rate and high social costs [2–6].

The incidence of these fractures is expected to rise from 1.26 million in 1990 to 4.5 million in 2050 [2]. The mortality rate caused by hip fractures in older adults is between 20% and 35% [7], and the female sex is more affected than the male sex.

The risk factor assessment on admission to the emergency department (ED) is critical to evaluate and possibly prevent the mortality of the patients after surgery [3, 8]. Preoperative conditions, including surgery timing, diabetes, cardiovascular disease, and pulmonary disease, are related to mortality [9].

Similarly, frailty among older adult patients is independently associated with mortality, irrespective of other clinical factors [10]. Predictive models, such as the Nottingham Hip Fracture score (NHFS), are very useful in stratifying mortality risk [11], estimating 30-day mortality and 1-year mortality in patients with femoral neck fracture [12], and assessing mobility grade 3 months after surgery and hospitalization length [13]. Another prognostic model is the American Society of Anesthesiologists score (ASA score) [14], which evaluates patient’s preoperative health status. The ASA score may be useful in clinical practice since studies have shown it to be effective in predicting 1-year mortality in patients with femoral neck fractures [15]. However, the NHFS shows better prognostic accuracy than the ASA score in predicting mortality within 30 days after surgery [13]. The Charlson Comorbidity Index (CCI) is a score created in 1987 to estimate mortality within 1 year and assigns severities to 19 comorbidities [16]. In 1994, age was introduced as a determinant of mortality. A high score of CCI in patients with hip fracture is associated with an elevated rate of admission in intense care units and postsurgical complications. The age-adjusted Charlson Comorbidity Index (aCCI) is particulary relevant in hip fractures, with a 31% increase in mortality by one unit increase in aCCI [17].

Patients with proximal femur fractures often have multiple comorbidities, with many having more than three [18–20], increasing the risk of both intrahospital and community-acquired infections, such as pneumonia, urinary tract infections, sepsis, and surgical site infections, within 30 days and 1 year postfracture [21]. Additionally, a higher comorbidity burden correlates with increased mortality rates, particularly in patients with more than three comorbidities, who experience a higher risk of death within 1 year compared with those with fewer comorbidities [22]. Preexisting conditions such as congestive heart failure, ischemic heart disease, dementia, chronic kidney disease, and cancer are also linked to higher mortality rates up to 2 years after the fracture [16].

Several studies in literature show that accurate preoperative assessment of the patient and stabilization of the clinical condition are important to improve outcomes [6, 23, 24].

The American Academy of Orthopedic Surgeons recommends surgery for proximal femur fractures within 48 h, as it is associated with reduced mortality [25]. Studies in literature show that early surgery, particularly within 24 h, reduces 1-year mortality and postoperative complications [26]. There is a proportional relationship between surgical timing and mortality risk; notably, early intervention within the first 24 h is critical [27]. Moreover, evidence from *Injury, International Journal of the Care of the Injured *(2019) [28] indicates an association between delayed surgery and increased 30-day mortality among patients with hip fracture with no or moderate comorbidities, contrasting with findings in those with higher comorbidity levels.

Our study aimed to identify clinical factors predicting hospital mortality in older adults with hip fractures admitted to the ED, with a particular focus on the relationship between surgical timing and acute and preexisting comorbidities.

The primary study endpoint was to evaluate risk factors associated with hospital mortality. As a secondary endpoint, we assessed the relative weight of factors predisposing patients to mortality, such as clinical and physiological parameters, and also performed a second analysis on the basis of surgical waiting time.

## Materials and methods

### Study design and inclusion/exclusion criteria

This is a single-center, retrospective observational study. All patients older than 65 years admitted to our emergency department (ED) from January 2018 until October 2023 with a diagnosis of proximal femur fracture (medial and lateral) classified according to an "Arbeitsgemeinschaft für Osteosynthesefragen" (AO) classification such as AO 31 A or 31B and requiring surgical treatment were recruited. Patients on anticoagulant therapy were included in the study, although their surgical treatment may have been delayed owing to the need for medication adjustment and management. We excluded patients under 65 years old, those who were transferred to other institutions, and those who refused hospitalization, and we did not apply any further age-based exclusions. We also ruled out data from March 2020 to April 2021 since they, particularly the surgical timing, would have been mostly affected by the coronavirus disease 2019 (COVID-19) pandemic [29]. The informed consent was waived owing to the anonymized retrospective design of the study. The study was conducted according to the principles expressed in the Declaration of Helsinki and its later amendments. The research protocol was approved by the Institutional Review Board of Fondazione Policlinico Universitario “A Gemelli” IRCCS—Rome (no. 0025817/22; study ID: no. 5121))*.*

### Study variables

All patients attended an initial preoperative evaluation consultation and underwent routine blood testing before surgery, including complete blood count, basic blood chemistry, coagulation studies, electrocardiogram, and chest X-ray upon admission to the emergency department (patients with abnormal findings in these tests underwent further specialized investigations). The blood tests performed before surgery were repeated on the first and third postoperative days and then repeated according to patients’ necessities. In this study, we considered electrolyte imbalance as the variations of electrolyte levels within body fluids; the reference values ​​in our hospital are sodium 135–145 mEq/l, potassium 3.5–5 mEq/l, calcium 8.5–10.5 mEq/l, and chlorine 95–105 mEq/l. We followed anticoagulation management preoperative protocols according to Italian Society of Orthopedics and Traumatology (SIOT) and Italian Society of Anesthesia Analgesia Resuscitation and Intensive Care (SIIARTI) guidelines before surgery. 

The global clinical evaluation analyzed the following variables: age, sex, clinical presentation (including the main symptoms at the ED admission), and preexisting comorbidities as defined by the Charlson protocol [30]. Using the physiological variables recorded on ED admission, we also calculated the NHFS for each patient. The clinical parameters were collected and recorded consistently throughout the entire study period.

The surgical indication was decided by experienced orthopedic surgeons according to the fracture type: 31 A type (femoral fractures involving trochanteric region) were reduced on the fracture table through traction and internal rotation of the leg injured and treated with intramedullary nailing, interlocked distally. Whereas 31B types (which are neck fractures) were treated with the same surgical approach (posterolateral approach) with hemiarthroplasty in case of patients over 80 years old with osteoporosis and with total hip replacement in case of under-80-year-old active patients with good functional requests. We did not cement implants in either case.

We followed standard protocols of our hospital for infection prevention, and all enrolled patients received a single dose of prophylactic antibiotic therapy 30 min before surgery. This one-shot antibiotic therapy was selected on the basis of local antibiograms and institutional guidelines. Prolonged antibiotic therapy was only administered if a postoperative infection was diagnosed, on the basis of microbiological cultures and clinical findings. In these cases, antibiotic therapy was tailored to target identified pathogens to ensure optimal therapeutic outcomes.

The criteria of the National Center for Infectious Diseases (Centers for Disease Control and Prevention, USA) were used to document periprosthetic infection [31]. Infections at the incision site or deeper underlying tissue that arose within 30 days of a surgical surgery were classified as superficial wound infections [32]. For suspected deeper infections, additional diagnostic procedures, such as imaging studies (e.g., CT scans or ultrasound) and/or tissue biopsies, were performed as needed. Infections were treated with tailored antibiotic therapy based on the results of microbiological cultures from biopsy samples or fluid aspiration.

A respiratory infection was defined as a positive sputum culture or a positive chest X-ray that looked like pneumonia or pleural effusion [33].

Changes in urinary nitrite, leukocyte blood count, or microbe presence on urine culture were used to identify urinary tract infections [34]. A significance threshold of ≥ 10^5^ CFU/mL is typically used for noncatheterized samples. We used quick Sequential Organ Failure Assessment score criteria (qSOFA): altered mental state, a systolic blood pressure of less than 100 mm Hg, and a respiration rate of 22 breaths per minute or higher. Sepsis was identified in those patients who satisfied two out of the three qSOFA [35].

### Statistical analysis

Continuous variables were reported as median (interquartile range) and categorical variables as numbers (percentage). In case of missing laboratory parameters, data were imputed using the Monte Carlo method. Imputation was performed only in patients with a maximum of two missing parameters.

The variables were compared at univariate analysis by the Mann–Whitney *U* test and the chi-squared test, as appropriate. Significant variables at univariate analysis were entered into a logistic regression model to find the independent predictors for in-hospital death. Separate logistic regression models were used to find the variables associated with poor outcomes in the group of patients surgically treated < 24 h and in the group with higher surgery timing.

In these two groups, the neural network analysis was used to evaluate the relative weight of the studied variables for the occurrence of in-hospital death. The neural network analysis was based on a multilayer perceptron procedure, using the study variables as predictors and the in-hospital mortality as the target variable. Each dataset was divided into training and testing samples with a 70% and 30% proportion, respectively. A single hidden layer was chosen over a multilayer approach to simplify the model and mitigate the risk of overfitting given the dataset size. Additional complexity introduced by multiple layers may not have been justified by available data size and could have increased model training time without significantly enhancing predictive performance. The analysis was carried out separately for the patients with a surgical time shorter and longer than than 24 h. The variable weights obtained were standardized to facilitate the comparison (the relative value of each item assigned by the analysis was expressed as a percentage, assuming 100% for the highest). A two-sided *p*-value of 0.05 was regarded as significant in all the analyses. Data were analyzed using IBM SPSS statistics for Windows, Version 26 (IBM Corp. Armonk, NY, USA).

## Results

Overall, 2320 patients met the inclusion criteria and were recruited for the study. Of these, 1391 patients (60%) underwent surgery within 24 h, while 929 patients (40%) were treated after 24 h.

Reflecting the epidemiological prevalence of fractures among women, 73% of patients (1693) were female, and 27% (627) were male (Table 1).

**Table 1:** Demographic data

	Patients treated surgically within 24 h (percentile 25–75%)	Patients treated surgically after 24 h (percentile 25–75%)
Mean age, years (*n*)	84 (76–89)	84 (77–89)
Sex (F/M)	1035/356	658/271
Hemoglobin (g/dL)	12 (11/13)	12 (10.6–13.3)
White blood cells (10^9^/L)	10.5 (8.5–12.5)	10.3 (8.2–12.8)
Platelets (10^3^/L)	225 (183–280)	223(183–286)
Glicemia (mg/dL)	125 (108–152)	125 (108–156)
Creatinine (mg/dL)	0.8 (0.66–1.1)	0.8 (0.68–1)
Fibrinogen (mg/dL)	347 (295–427)	359 (292–448)
Waiting time before surgery (h)	13.1 (6–19)	32 (26–42)
Nottingham score (%)	2.37 (1.45–3.84)	2.37 (1.4–3.8)
Charlson Index (*n*)	4 (4–5)	4 (4–5)
Mortality (%)	2.8	5.2

Demographic and epidemiologic characteristics of the two-group population

The mean age was 84 years (range 77–89), with 100% of patients falling within this age range; the percentage of patients in each age interval (77–89 years) was consistent across both groups. The median NHFS was 2.37 (range 1.4–3.8). The two groups analyzed had comparable characteristics in terms of hemoglobin (HB), glycemia, creatinine, platelets (PT), and white blood cells (WBC).

The median waiting time before surgery for the first group, treated within 24 h, was 12.5 h (range 6–19), while for the second group, it was 32 h (range 26–42). Additionally, 106 patients (4.6%) were diagnosed with another acute medical condition in the emergency room, such as electrolyte imbalance, atrial fibrillation, or atrial flutter (AF).

Table 2 also lists chronic disorders such as heart failure, affecting 308 cases (13.3%); diabetes, affecting 193 cases (8.3%); and renal failure, affecting 69 cases (2.97%).

**Table 2:** Acute and chronic conditions data in the population

	Patients treated surgically within 24 h (%)	Patients treated surgically after 24 h (%)	Total of patients affected/total of patients (%)	Patients affected who died/total of patients who died (%)
Infection	44 (3)	64 (7)	108 (4.7)	19 (21.8)
Fever	17 (1.2)	36 (4)	53 (2.3)	7 (8)
Sepsis	3 (0.2)	8 (0.9)	11 (0.5)	7 (8)
Pneumonia	15 (1.9)	18 (1.4)	33 (1.4)	9 (10.3)
Acute events (atrial fibrillation, flutter, and electrolyte imbalance)	49 (3.5)	57 (6.1)	106 (4.6)	9 (10.3)
Stroke	5 (0.5)	5 (0.5)	10 (0.4)	2 (2.3)
Anemia	73 (5.2)	56 (6)	129 (5.6)	3 (3.4)
Heart failure	153 (11)	155 (16.7)	308 (13.3)	24 (27.6)
Diabetes	113 (8.1)	80 (8.6)	193 (8.3)	8 (9.2)
Kidney failure	30 (2.2)	39 (4.2)	69 (3)	12 (13.8)

Patients treated within or after 24 h, affected by different acute and chronic conditions

Among the 106 patients (4.6%) diagnosed with acute atrial fibrillation (AF) or electrolyte imbalance, 49 were treated within 24 h, and 57 were treated after 24 h (Table 2). Overall, 108 patients were diagnosed with a coexistent infection, including respiratory tract infections, urinary tract infections, superficial wound infections, and periprosthetic infections at the site of the fracture surgery. Of these patients, 19 (17.6%) died by the most recent follow-up period, contributing to a total mortality rate of 21.8% for the study population.

No more than 44 patients (3%) who suffered infections were treated within 24 h. The decision to treat infected patients before surgery was based on the severity and type of infection, as well as the patient’s clinical condition upon admission. Infected patients with mild symptoms or stable vital signs were typically treated after surgical intervention, while those with more severe infections requiring immediate intervention were prioritized for treatment before surgery. Sepsis was diagnosed preoperatively in 11 patients (0.47%), of whom, 4 (36.4%) remained alive at the latest follow-up. Only three of these sepsis cases (27.3%) were treated within 24 h.

Pneumonia was identified in 18 patients (1.9%) of the first group (treated within 24 h) and in 15 patients (1.4%) of the second group (treated after 24 h).

For patients who underwent surgery within 24 h, the in-hospital mortality rate was 2.8%, compared with 5.2% for those who underwent surgery after 24 h (*p* = 0.046; OR 1.58). Age (*p* = 0.001; OR 1.06) and Nottingham Hip Fracture Score (*p* = 0.04; OR 1.32) were factors predicting mortality, as shown in Table 3.

**Table 3:** Multivariate analysis of general data

	*p-*Value	Odds ratio (OR)	Inferior	Superior
Age	0.000	1.063	1.028	1.100
Charlson Comorbidity Index	0.062	1.185	0.991	1.417
Nottingham score	0.041	1.324	1.011	1.733
Surgery within 24 h	0.046	1.580	1.008	2.478
Acute events	0.118	1.870	0.853	4.100
Acute infections	0.000	5.992	3.315	10.832
95% confidence intervals (C.I.) per OR				

Factors predicting mortality

Acute infections were associated with a high risk of mortality during recovery (*p* = 0.001; OR 5.99), both in patients treated within and after 24 h (Table 4). Acute events, such as atrial fibrillation and electrolyte imbalance, were related to mortality risk only in patients treated within 24 h (*p* = 0.001 versus *p* = 0.51).

**Table 4:** Multivariate analysis (within and after 24 h waiting for surgery)

Surgery within 24 h	*p-*Value	Odds ratio (OR)	95% C.I. Inferior	95% C.I. Superior
Age	0.001	1.093	1.035	1.153
Charlson	0.487	1.109	0.828	1.486
Nottingham	0.106	1.390	0.933	2.070
Acute event	0.001	5.366	2.032	14.169
Acute infections	0.002	5.068	1.790	14.352
Age	0.038	1.046	1.003	1.092
Charlson	0.087	1.232	0.977	1.554
Nottingham	0.177	1.291	0.891	1.870
Acute event	0.517	616	0.142	2.670
Acute infections	0.000	6.253	3.009	12.994

Factors predicting mortality analyzed within and after 24 h waiting for surgery

**Fig. 1:**
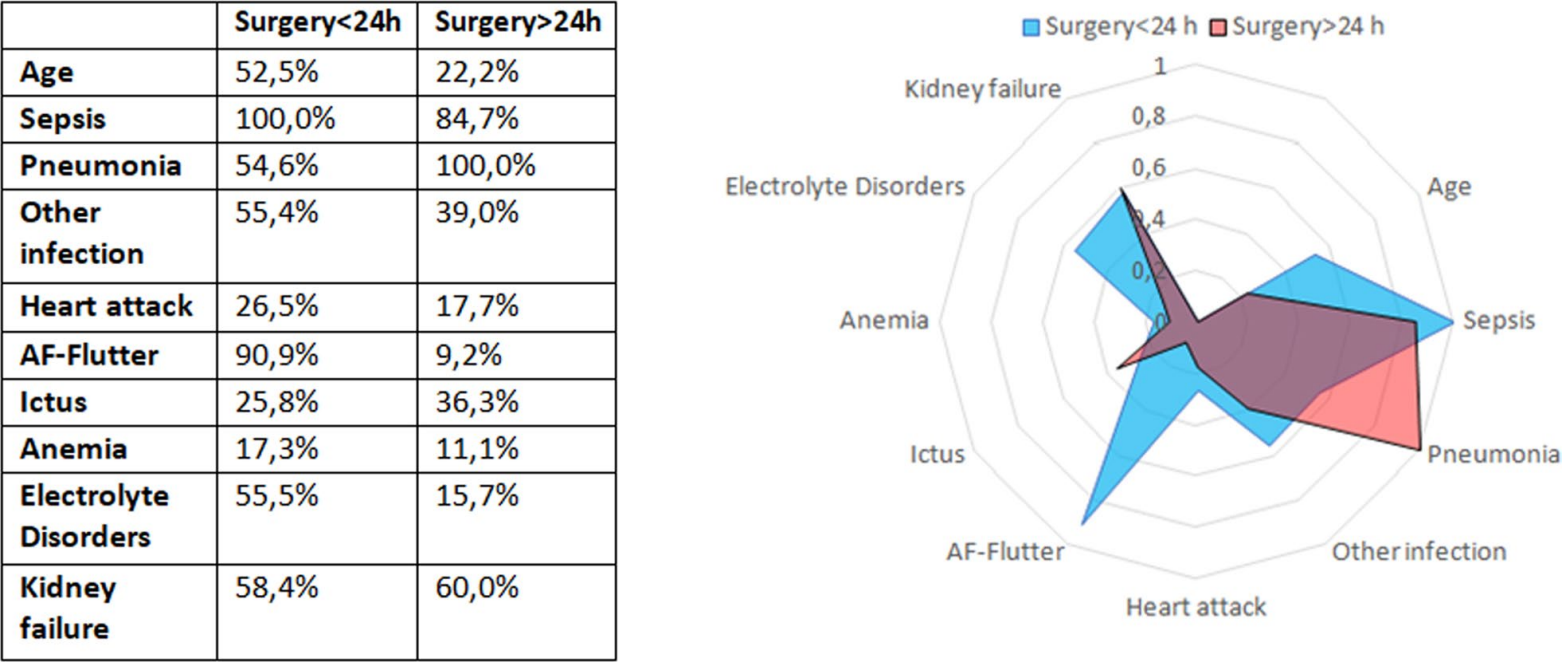
Neural network analysis comparison of mortality weights for variables in groups treated within 24 h and after 24 h

Figure 1 depicts the neural network analysis representation, which provides a mortality weight for each variable studied, comparing the first group treated within 24 h to the second group treated after 24 h. Acute events such as atrial fibrillation and flutter (90%), electrolyte imbalance (55%), acute infections (55.4%), and sepsis (100%) had the highest relative weight for in-hospital mortality in patients treated within the first 24 h. Conversely, chronic diseases such as kidney failure (60%) and pneumonia (100%) were most prevalent in patients who died after being treated beyond 24 h.

## Discussion

This study aimed to explore the effects of surgical timing on mortality and complications in older adults with hip fractures. Our main findings reveal that early surgery, performed within 24 h, is associated with a significantly lower mortality rate compared with surgery performed after 24 h (2.8% versus 5.2%, *p* = 0.046). However, we also found that acute infections and sepsis, regardless of the timing of surgery, are linked to increased mortality rates, suggesting that in these cases, prompt surgical intervention may be more beneficial than waiting for clinical stabilization.

Hip fractures are severe injuries that can result in postoperative complications, disability, lifelong immobility, and death, particularly in older adults [4, 36, 37]. Age, gender, comorbidities, anticoagulant medication, general health status, and the timing of surgery are all known factors that impact the survival of these patients. The relationship between the timing of surgery and postoperative outcomes has been widely studied and is known to influence the clinical outcomes and survival of patients suffering from hip fractures.

Although international clinical practice guidelines recommend surgical treatment of acute hip fractures within 48 h to prevent the occurrence of postoperative complications [26], in clinical practice, delay of surgery of hip fractures is common [4]. Most delays are caused by a patient’s comorbidities and acute events occurring at arrival in the emergency room, which increases surgical risk and perioperative complications, such as pneumonia, deep venous thrombosis, hemorrhage, pulmonary embolism, urinary tract infections, and decubital ulcerations [4].

Generally, clinicians take time to optimize patients’ medical conditions before surgery, even though some studies show that early surgery is connected to a shorter length of stay, less discomfort, a decrease in significant complications, and a drop in death rates for patients. [26, 38].

The timing–outcome relationship has been previously demonstrated in many studies: early surgery is beneficial in clinical outcomes and patient survival [27]. For example, Maheshwari et al. [27] illustrated a linear relationship between the delay in surgery and mortality. With every 10-h increase in time from hospital admission to surgery, there is an estimated 5% increase in odds of 1-year mortality. In our results, the mortality rate in patients that we treated within 24 h was 2.8%, and for those who underwent surgery after 24 h, the mortality was 5.2% (*p* = 0.046; OR 1.58), corresponding with findings from literature. There are numerous studies in literature with medium and long-term follow-ups that show a significant increase in the risk of mortality related to surgical timing [39–41].

According to epidemiological statistics from Pinto et al., older individuals with proximal femoral fractures have a 1-year death rate ranging from 14% to 20% [41], with some studies reporting an even higher rate of 40% [42].

This worrying fatality rate stresses the importance of identifying risk factors for death following surgery: age, gender, preoperative functional capability, preexisting comorbidities, and general health status [43]. While there has been extensive research on mortality predictors, there is limited exploration of risk prediction models and preventable risk factors. As mentioned, numerous studies agree that receiving surgical treatment as an urgent procedure, once the body meets the basic anesthetic requirements, is beneficial for older adults [44, 45], and lots of orthopedic societies such as the American Academy of Orthopedic Surgeons (AAOS) recommend surgical repair within 48 h of admission [45].

These findings emphasize the critical implications and potential complications associated with hip fractures in older adults, highlighting the significance of early and appropriate medical care to improve outcomes and reduce mortality risk. Unfortunately, mechanisms for how early surgery decreases mortality are still unclear, and the reasons for this association remain unanswered. According to Maheshwari et al., the prolonged inflammatory response in delayed surgery can lead to an increased risk of infection, delirium, and cardiovascular complications, which increase mortality risk. [27]. In 2017, Daniel Pincus conducted a study that included 42,230 patients with a mean age of 80.1 years treated surgically for hip fracture. The study showed that patients who postpone the operation by 24 h suffered complications such as pneumonia (*p* = 0.002), pulmonary embolism (*p* < 0.001), and myocardial infarction (*p* < 0.001) more frequently when compared with those whose waiting time before surgery was less than 24 h [46]. 

Furthermore, Dong et al. [26] performed a comprehensive review in 2011 including 15 studies, which showed that the occurrence of postoperative complications was influenced by the waiting time: patients who took more than 48 h to undergo surgery experienced severe postoperative complications and suffered not only pneumonia, pulmonary embolism, and myocardial infarction but also sepsis (*p* = 0.008) and cardiac arrest (*p* = 0.039) when compared with patients treated within 48 h (*p* = 0.010; *p* = 0.043) [26].

The most frequent postoperative complications described are confusion, urinary tract infection, and pneumonia (especially in people affected by pulmonary diseases) [21, 47]. In addition, surgical-site infections that require reoperation are of significant complication owing to the severity and complexity of treatment, and, probably, the association between poor wound healing and infection underlies a biological mechanism related to preexisting morbidities such as diabetes [21]. Indeed, comorbidities are important risk factors not only for surgical problems but also for postoperative complications, affecting patient outcomes; they are connected to infection up to 1 year after surgery [21].

In the study of Gadgaard et al., within 1 year after surgery, the cumulative incidence of any hospital-treated infection was 22.2% among patients with no comorbidity, 29.8% among patients with moderate comorbidity, and 36.6% among patients with severe comorbidity, showing that the cumulative incidence of any hospital-treated infection increased with increasing comorbidity level [21]. Our study results reveal a different aspect of this picture: acute infections and sepsis are related to increasing mortality rates in both patients treated surgically within 24 h and after 24 h, without significant differences. It suggests that treating patients with acute infections and sepsis surgically does not change the outcome in relation to surgery timing. In such cases, waiting for their full clinical recovery and reconditioning does not improve the postsurgical survival rate.

Furthermore, delaying surgery in the hope of clinical recovery and reconditioning might not yield better outcomes, as the ongoing presence of an acute infection or sepsis can lead to further deterioration of the patient’s condition. Instead, a timely surgical approach appears to be a good way of managing these high-risk patients effectively.

Our findings suggest that infections not only elevate mortality rates in individuals with significant comorbidities who are most susceptible but also impact patients in generally healthier conditions (OR 5.992). According to our results, the correlation between the Charlson Comorbidity Index (CCI) and mortality was not statistically significant (*p* = 0.62). Unlike the findings in literature, our results indicate that the CCI did not have a significant impact on mortality because it pertains to chronic diseases and comorbidities rather than the acute conditions that can coexist with fractures.

Conversely, acute events such as atrial fibrillation, flutter, and electrolyte depletion appear to destabilize patients’ conditions and seem to have a greater impact on their outcomes. In our study, neural network analysis revealed that sepsis (10%), acute infections (55.4%), electrolyte imbalances (55%), and atrial fibrillation and flutter (90%) had the greatest relative significance regarding the risk of mortality in patients treated within the initial 24 h.

Indeed, acute events were related to increased mortality risk, particularly in patients treated within 24 h. This observation suggests that the urgency of treating acute events necessitates immediate medical intervention to stabilize the patient before surgery. Stabilization is crucial to minimize intraoperative and postoperative complications, which can otherwise exacerbate the patient’s condition and increase the likelihood of mortality.

NHFS is a risk assessment system designed exclusively for patients with hip fracture. It determines the preoperative mortality risk within 30 days of a hip fracture and is also related to postoperative mobility and hospitalization rate [13]. NHFS association with mortality, similar to CCI, was not statistically significant, most likely for the same reasons we discussed above.

Our study did not extensively explore comorbidity as a risk factor for post-hip fracture surgery infections. Additionally, our focus was not on a specific type of infection, as it was not the primary endpoint of our study. Furthermore, rehabilitation, complications, and pharmacologic treatments after hospital discharge were not collected.

### Study limitations

Several limitations must be acknowledged in this study. First, the retrospective design introduces potential biases, including selection bias and information bias, as data were extracted from existing medical records. This approach limits our ability to control for all potential confounders and restricts the ability to establish causal relationships. Second, the study focused on a limited number of perioperative complications such as surgical timing, in-hospital mortality, and a few complications, excluding other potentially influential factors such as postoperative rehabilitation, long-term functional outcomes, or specific comorbidities. These additional factors could have significantly impacted the results but were beyond the scope of this study.

Moreover, the sample population consisted primarily of older adults, which may limit the generalizability of our findings to younger individuals or those with different clinical characteristics. The variability in patient management protocols across the study sites also introduces another limitation. Inconsistent preoperative, intraoperative, and postoperative care across sites was not accounted for and could have influenced the outcomes.

Additionally, data on post-discharge outcomes, including long-term rehabilitation, functional recovery, or quality of life, were not collected. These factors are crucial for evaluating the comprehensive impact of surgical timing on patient health and recovery. Finally, although efforts were made to control for known variables, other unmeasured confounders, such as the type of surgical procedure or anesthesia used, may have influenced the results. Future studies with a prospective design, larger sample sizes, and more detailed data collection would help address these limitations and provide a deeper understanding of the relationship between surgical timing and patient outcomes in hip fracture surgeries.

## Conclusions

Hip fractures are the most common orthopedic injury in older adults, significantly contributing to morbidity, mortality, and high social costs. The timing of surgical treatment is known to be crucial for postoperative outcomes, and early surgery is essential to reduce the postoperative mortality rate. While mortality decreases if surgery is performed within 48 h, multiple comorbidities often lead to delays in surgery in order to stabilize the patient’s clinical condition.

Our study demonstrates that, while early surgery is essential for patients without acute medical complications, stabilizing the patient before surgery may improve outcomes for those with acute events such as electrolyte imbalances, atrial fibrillation, or flutter. In these cases, a delay in surgery may lead to better postoperative tolerance and improved outcomes. However, we found that acute infections and sepsis are associated with increased in-hospital mortality rates regardless of whether surgery is performed within or beyond 24 h. Thus, these conditions may require prompt surgical intervention, rather than a delay, to minimize their impact on in-hospital mortality.

## Data Availability

All of the data that we analyzed and tables we compiled are available for any clarification.

## Abbreviations

ED: Emergency department
NHFS: Nottingham Hip Fracture Score
ASA score: American Society of Anesthesiologists score
CCI: Charlson Comorbidity Index
aCCI: Age-adjusted Charlson Comorbidity Index
qSOFA: Quick Sequential Organ Failure Assessment score criteria
AAOS: American Academy of Orthopedic Surgeons
